# Evidence-based therapeutic management of binge-eating disorder

**DOI:** 10.1192/j.eurpsy.2021.941

**Published:** 2021-08-13

**Authors:** D. Vasile, O. Vasiliu

**Affiliations:** Psychiatry, University Emergency Central Military Hospital Dr. Carol Davila, Bucharest, Romania

**Keywords:** binge-eating disorder, psychotherapy, lisdexamfetamine, antidepressants

## Abstract

**Introduction:**

Binge-eating disorder (BED) is a difficult-to-manage clinical entity, that may associate both organic (e.g., obesity, metabolic syndrome) and psychiatric (e.g., anxiety, mood, or substance use disorders) co-morbidity. Psychotherapeutic and pharmacological approaches are usually combined in order to reach the best outcome for these patients, but the disorder seems to have prolonged evolution even under appropriate therapeutic managment.

**Objectives:**

To evaluate the most evidence-based therapies focused on BED.

**Methods:**

A literature review was conducted through main electronic databases, and papers published between January 2000 and August 2020 were included in the analysis.

**Results:**

Cognitive-behavioral therapy (CBT) is supported by multiple trials, and it led to decreased number of binge episodes and increased rates of remission. Behavioral activation may improve certain symptoms of BED (depressive mood, anxiety), but not the binge episodes frequency. Interpersonal group therapy (IPT) may be helpful for BED patients with an external locus of control and significant interpersonal dificulties. Dialectical behavior therapy (DBT) has been applied in BED patients, but the results have been inconclusive. Lisdexamfetamine dimesylate is the only FDA approved drug for this indication, as dasotraline was rejected by FDA and its research discontinued by the manufacturer. Fluoxetine, sertraline, escitalopram, duloxetine, bupropion, atomoxetine, reboxetine, armodafinil, disulfiram, baclofen, zonisamide, lamotrigine, topiramate, samidorphan, liraglutid, and orlistat need more trials in order to validate their efficacy, especially on long term.

**Conclusions:**

There is only one drug currently FDA approved for this indication, lisdexamfetamine, and a number of psychotherapies, with CBT and IPT being the most supported by evidence.

